# The Book World of Medicine and Science

**Published:** 1900-06-02

**Authors:** 


### The Book World of Medicine and Science.

Plea for a Simpler Life, and Fads of an Old Physician. By George S. Keith, M.D., LL.D., F.R.C.P.E. (London: Adam and Charles Black. 1900. Price 3s. 6d.)

As may be seen from its title, this is the book of an old man and a faddist, and it partakes of both the virtues and the vices natural to such a production. It is egotistical to a degree; it is full of self-glorification at the expense of other medical men; it is laden with scorn at what the writer seems to suppose are the teachings of the medical profession; and it contains a good deal of nonsense about the tyranny of medicine, and the advantage of people treating themselves. Yet, underlying it all, there is a grain of truth, and, by dint of grinding away at this grain, and girding at "the doctors"—and what is there in a semi-medical work that can possibly give more pleasure to the ordinary reader than a good slap at the doctors?—Dr. Keith has managed to make a very readable although rather monotonous book. The little grain of truth, which he has so ingeniously ironed out to fill his book, is that we all of us eat too much, and that when the stomach cannot digest food, food is best withheld, a view which does not seem to us overburdened with novelty. Dr. Keith, we think wisely, addresses the public rather than the profession. In fact, to the profession his book tells little. Still, a good many cases are mentioned which are not without interest as illustrations of two well-known facts: first, that one of the worst foes of the dyspeptic is appetite, leading him, as it does, to overburden his enfeebled stomach; and, second, that most people carry a large reserve of nutrient material stored up in their tissues which is perfectly available as "food" if the wants of the body in regard to water are kept supplied. The importance of some of the cases from a medical point of view lies in the proof they give that the utility of the so-called starving plan is not confined in any way to fat people. Dyspepsia is a malady in which toxæmia and the discomfort resulting from the fermentation plays so large a part that the almost total abstinence from food affords much temporary relief, while giving time for the digestive organs to recover their power. Thus are to be explained some of the cases, striking enough in themselves, related by Dr. Keith in which people who were at the lowest ebb still managed to come round when they were kept warm and quiet and fed with hot water and the smallest possible quantity of food, generally skim-milk in quite small doses, such as a wine-glassful three times a day. In regard to alcohol Dr. Keith's remarks go to show that craving for it is increased by meat diet, and that when people can be got on to a simpler and more largely vegetable dietary the craving for stimulants becomes less. His remarks on high feeding and the morals of the young are also of some importance. There can, indeed, be but little doubt that in some cases it is only by dint of constant athletics that the animal tendency of too much animal food is controlled. There is so much that is good in this book that we could have wished that its author had exalted himself a little less at the expense of his brethren.

On Granular Kidney and Physiological Albuminuria: Being the Lettsomian Lectures delivered before the Medical Society of London by Samuel West, M.A., M.D. Oxon., F.R.C.P. (London: Henry J. Glaisher. 1900. Price 7s. 6d. net.)

As read before the Medical Society, these lectures appeared to treat their subject in an orderly manner. The same may be said of the book before us, if one follows the text and misses out the headings. But never, we should think, was a book so chopped up and its order disturbed by divisions and sub-divisions as this is. Dr. West begins by showing the frequency and importance of the disease which goes by the name of granular kidney, the forms it takes, and its relations to acute nephritis and arterial disease. In regard to the much-vexed question of the connection between granular kidney and acute nephritis, he shows that however clear it may be that the latter may end in a large white kidney, and this again in a contracting white kidney, while this again may possibly end in a contracted white kidney, which is practically the same as one of the forms of cirrhosis met with in granular kidney, there is a weak link in the chain between the *contracting* white and the *contracted* white, and he urges with great force that so slight are some of the early symptoms of granular kidney, and so common are intercurrent attacks of acute mischief in its course, that even in the comparatively rare cases in which granular kidney can be shown to have followed acute nephritis, there is much probability that the more chronic disease had already been in progress before the acute attack which is assigned as its cause. Physiological albuminuria is then dealt with, the conclusions being arrived at: (1) That the so-called physiological albuminuria is always pathological, even if not always renal, when the amount of albumin is more than the merest trace; (2) that it is probably pathological even in those cases in which the amount of albumin is but a trace, and no cause obvious. Much interesting information is given in regard to albuminuric retinitis, the toxæmic symptoms met with in granular kidney, and the skin affections which are apt to develop in its course. Treatment also is not neglected, and, taken altogether, the book is a most valuable and complete description of a morbid condition the ramifications of which extend far more widely than its name implies.

